# A hybrid high activity aware framework integrating graph attention network and transformer for half maximal inhibitory concentration prediction

**DOI:** 10.3389/fimmu.2026.1929215

**Published:** 2026-08-26

**Authors:** Dingcheng Ban, Lu Pan, Xueli Zhang, Peng Xu, Binbin Wang, Tao Liu, Deng Pan, Xianbin Li

**Affiliations:** 1 School of Computer and Big Data Science, Jiujiang University, Jiujiang, China; 2 Jiujiang Key Laboratory of Digital Technology, Jiujiang University, Jiujiang, China; 3 Institute of Management and Health (IMH) Swansea Business School, University of Wales Trinity Saint David, Swansea, United Kingdom; 4 Department of Medical Technology, Zhengzhou Railway Vocational and Technical College, Zhengzhou, China; 5 Institute of computational science and technology, Guangzhou University, Guangzhou, China

**Keywords:** c-kit, graph attention network, half maximal inhibitory concentration prediction, high activity aware, transformer

## Abstract

**Introduction:**

Tyrosine kinase inhibitors targeting the c-KIT receptor are pivotal in the targeted therapy of malignancies such as gastrointestinal stromal tumors (GIST). The bioactivity of these inhibitors is typically quantified by the half-maximal inhibitory concentration (IC_50_), making its accurate prediction a critical computational task for accelerating the discovery and optimization of anticancer lead compounds.

**Methods:**

To address this need, we propose HGATT—a hybrid high activity aware framework integrating Graph Attention Network (GAT) and Transformer—for high-accuracy half maximal inhibitory concentration (IC_50_) prediction of c-KIT inhibitors. The model was trained on inhibitor data targeting c-KIT and related kinase families sourced from the BindingDB and ChEMBL databases. By extracting atom-level graph features, Morgan fingerprints, and physicochemical descriptors from SMILES strings, HGATT constructs a unified molecular representation that integrates both local structural and global information. Its architecture employs multidimensional graph attention mechanisms and gated residual modules to simultaneously capture atomic-level local structural features and macroscopic molecular properties. Stabilized training strategies, including gradient clipping, were adopted to enhance training efficiency and model robustness.

**Results:**

On an independent test set, HGATT achieved a mean squared error (MSE) of 0.28 and a coefficient of determination (R²) of 0.57, corresponding to an approximate 44% reduction in MSE compared to the second-best baseline.

**Discussion:**

Experimental results demonstrate that HGATT outperforms not only individual graph neural network (GNN)- and machine learning-based models but also other related drug-target prediction methods and baseline regression approaches, exhibiting superior predictive accuracy.

## 1 Introduction

c-Kit, a classic proto-oncogene at 4q12, is activated by ligand-induced dimerization and autophosphorylation, triggering the PI3K/AKT, MAPK/ERK, and JAK/STAT pathways. These pathways regulate a spectrum of cellular behaviors, from normal proliferation and differentiation to tumorigenesis, migration, metastasis, and recurrence (1). Research indicates that oleanolic acid specifically inhibits c-Kit expression and downregulates key components within the c-Kit-mediated PI3K/AKT and MAPK/ERK signaling cascades (2). Consequently, tumor proliferation is suppressed through the induction of cell-cycle arrest (primarily in the G0/G1 phase) and the promotion of mitochondrial-dependent apoptosis (3). However, all currently approved c-Kit-targeting agents exhibit off-target activity, which frequently leads to adverse events—including anemia, neutropenia, hemorrhage, hypertension, and diarrhea—and collectively contributes to the development of drug resistance (4). Therefore, the development of novel, safe, and selective c-Kit inhibitors remains an urgent priority in oncology therapeutics.

In recent years, computer-aided drug design (CADD) has accelerated the discovery of lead compounds through the application of molecular modeling and computational simulation. The increasing availability of high-resolution three-dimensional structural data has further advanced structure-based CADD approaches (5). Complementarily, ligand-based drug design (LBDD) develops structure-activity relationship (SAR) or pharmacophore models by leveraging known active compounds, with representative methods including quantitative structure-activity relationship (QSAR) modeling, pharmacophore modeling, receptor mapping, and molecular shape alignment (6). Using the QSAR method, Chaudhari et al. (7) identified five molecules from the ZINC database as potential c-KIT inhibitors, demonstrating high predicted in silico activity and strong key binding interactions with the c-KIT receptor. Keretsu et al. (8) performed computational studies on 48 pyrazolopyridine derivatives, which exhibited inhibitory activity against both c-KIT and PDGFRα, with the aim of elucidating the structural properties critical for dual-kinase inhibition. Almerico et al. (9) employed a three-dimensional quantitative structure-activity relationship (3D-QSAR) method, aimed at identifying 95 c-Kit tyrosine kinase inhibitors. For a long time, QSAR models have dominated this field. However, their performance heavily relies on manually designed molecular descriptors, making it difficult to effectively capture the complex nonlinear mapping relationships between molecular structures and biological activity.

Another computational approach is based on graph neural networks (GNNs). GNN technology naturally represents molecules as graph structures, where atoms serve as nodes and chemical bonds as edges. These elements are encoded directly into vectors using fundamental physicochemical properties. This enables end-to-end molecular feature learning and opens new avenues for IC_50_ prediction. For example, Chen et al. (10) proposed SGNet, a sequence-based convolution and ligand graph network that integrates molecular graph and amino acid sequence data. The model extracted protein features via Conjoint Triad (CT) encoding and a 1D-CNN, captured ligand features with a graph attention network, and fuses both representations through a fully connected layer to predict binding affinity. Zhang et al. (11) presented GraphTCDR, a heterogeneous graph neural network that leverages multi-omics data and drug features to predict cancer drug response. The model operated by building a cell line–drug heterogeneous graph, learning node representations from it, and ultimately predicting IC_50_ values through fully connected layers. Wang et al. (12) introduced a fingerprint-enhanced graph attention convolutional network (FnGATGCN) for predicting the activity of anti-NSCLC drugs. Using a multimodal fusion approach, the model incorporates a feature extraction layer that combines molecular graph features with molecular fingerprint features. Rong et al. (13) employed molecular graphs as an advanced approach for peptide characterization and applied a graph convolutional network (GCN) model to predict variable-length peptides. Karlov et al. (14) presented a graph-convolutional neural network (GCN) model designed to predict the binding constants of protein-ligand complexes. Ji et al. (15) applied a hybrid virtual screening pipeline that integrated ligand-based and structure-based methods to identify small-molecule candidates targeting GluN1/GluN3A. The pipeline employed a graph neural network-based drug-target interaction model to improve docking precision. Nevertheless, when facing the specific challenge of screening highly active c-KIT inhibitors, existing GNN methods still exhibit significant shortcomings. On the one hand, standard graph attention networks (e.g., GAT), while capable of aggregating neighborhood information, lack a specific attention mechanism for activity-relevant substructures such as key pharmacophores, resulting in limited ability to distinguish highly active molecules. On the other hand, most models rely on a single source for molecular representation and fail to synergistically utilize multi-scale molecular information, which caps their representational capacity. More critically, these models generally do not incorporate a dynamic weighting mechanism for high-activity regions, making it difficult for them to prioritize the identification of complex molecular structures with ultra-high inhibitory potential in virtual screening, thereby lacking sufficient sensitivity and practicality.

To address the aforementioned bottlenecks, we propose a hybrid high activity aware framework that integrate GAT and Transformer for IC_50_ prediction (HGATT). The framework mainly lies in the synergistic design of multi-source feature fusion and activity-guided attention mechanisms. Specifically, the model constructs a unified and rich molecular representation by integrating molecular graph structures, global descriptors, and local fingerprint information. Furthermore, an activity-score-driven gated attention mechanism is introduced to dynamically adjust the importance weights of different feature dimensions and molecular substructures, enabling the model to preferentially focus on key chemical features closely associated with high activity. During training, we employed strategies such as gradient clipping, residual connections, and stabilized training, significantly enhancing the model’s convergence speed, training stability, and generalization capability. Subsequent experimental results demonstrate that our method significantly outperforms various baseline models on the task of predicting IC_50_ for c-KIT inhibitors, validating its high efficiency and reliability, and providing a powerful computational tool for targeted drug development. We evaluated the proposed model on a c-KIT inhibitor dataset (containing 4,856 unique molecules) sourced from ChEMBL and BindingDB. The experimental results show that its performance is significantly superior to that of various mainstream baseline models. The contribution of this work lies in proposing a high-performance IC_50_ prediction framework and, through interpretability analysis, revealing the key structural features of highly active molecules, thereby providing a reliable computational tool for targeted drug discovery.

To sum up, the contributions of the proposed methods are listed as below:

1. HGATT accurately predicts c-KIT inhibitor IC_50_ (R²=0.57, MSE = 0.28);
2. A multi-scale molecular representation integrates graph, fingerprint and descriptor features;
3. An activity-aware attention mechanism dynamically highlights key pharmacophores.

## 2 Material and methods

**Figure 1:**
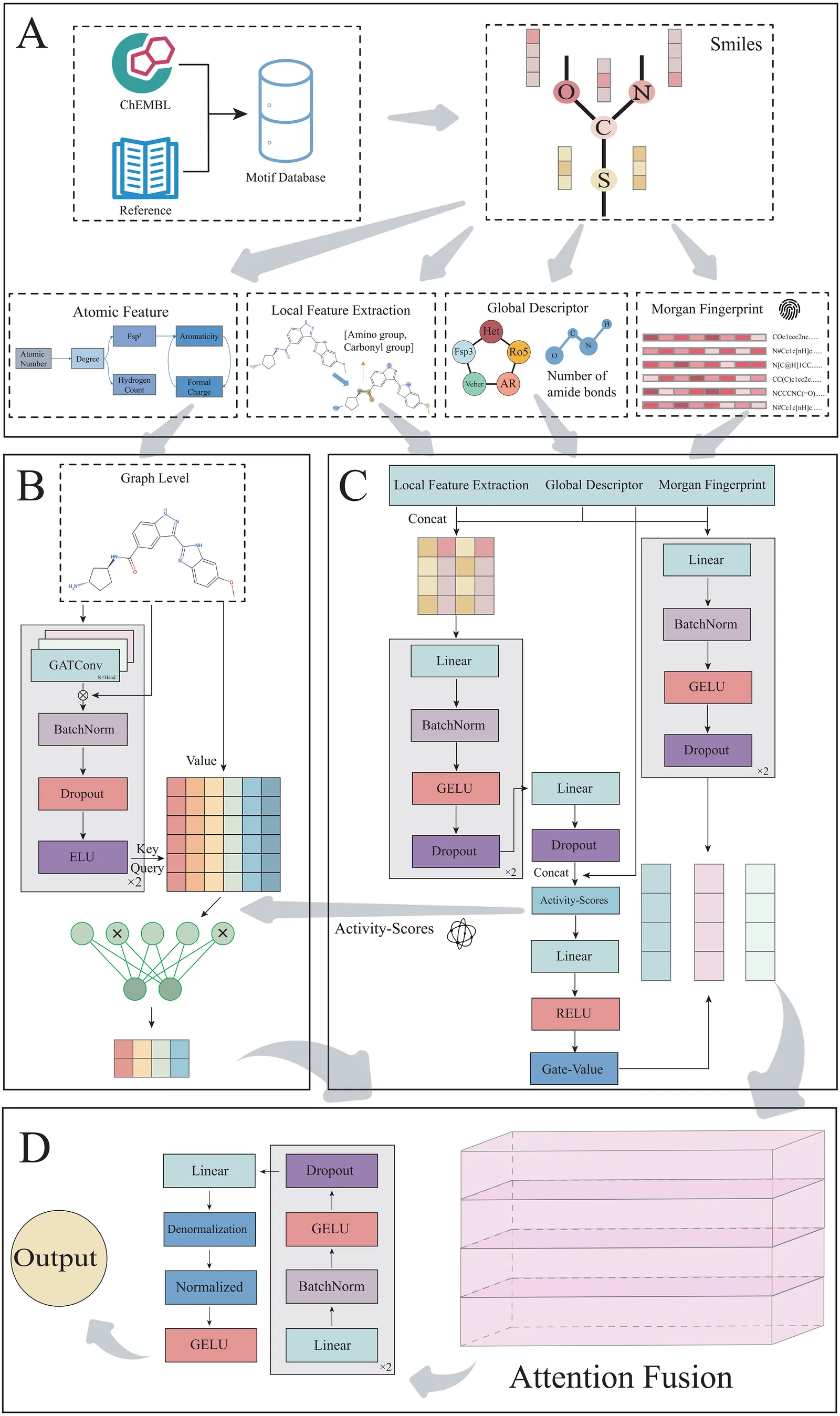
The workflow of this study. **(A)** Feature Extraction, **(B)** High Activity Aware Graph Encoder, **(C)** High Activity Aware Feature Projection, **(D)** High Activity Attention Fusion.

This study proposes a hybrid framework named the high activity aware molecular perception network, designed to achieve accurate prediction of molecular IC_50_ values. This section illustrates the preprocessing steps, including SMILES sequence parsing, Morgan fingerprint extraction, and global descriptor/local atom feature projection. It also details the activity-score-driven gated feature weighting mechanism. The entire workflow highlights the model’s specific design optimizations for high-activity molecules across key stages such as feature extraction, graph encoding, and multi-source fusion. As illustrated in **Figure 1**, the complete technical framework consists of four closely integrated core modules:

- Feature Extraction: First, global physicochemical descriptors (e.g., molecular weight, LogP) are computed to capture macroscopic molecular properties. Second, local atom features are extracted by representing each molecule as a graph, where atoms (nodes) are characterized by a 6-dimensional feature vector (including atom type, formal charge, etc.) and chemical bonds as edges, preserving atomic-level properties and topological connectivity. Third, Morgan fingerprints are generated to encode substructure-level chemical environments. These global descriptors, local atom features, graph structural information, and Morgan fingerprints are finally integrated into a unified multi-view representation, providing the model with comprehensive and complementary information for accurate activity prediction. The model takes multi-source molecular features (including SMILES sequences, atomic features, Morgan fingerprints, etc.) as input.
- High Activity Aware Graph Encoder: Employs a dual-layer GAT attention mechanism, enhanced with residual connections and activity-guided multi-head self-attention, to transform molecular graph structures into activity aware embeddings. This module utilizes a two-layer GATConv (Graph Attention Network) architecture. It incorporates residual connections and batch normalization, and introduces a multi-head attention mechanism based on activity scores to achieve the transformation from molecular graph data into activity-aware graph embeddings.
- High Activity Aware Feature Projection: This core module enhances molecular representation through a dual-branch architecture. The feature projection branch​ transforms input features into a latent space, while the parallel activity-aware gating branch​ uses an estimated activity score to compute adaptive weights. These weights are applied via element-wise multiplication, dynamically highlighting features most relevant to high bioactivity. To ensure stable and generalizable learning, the module integrates batch normalization​ after each linear transformation and employs dropout regularization​. Together, these components enable the model to focus on critical structural patterns associated with potent compounds while maintaining robust training performance.
- High Activity Attention Fusion: Integrates graph encodings, global descriptors, local atom features, and fingerprint information into a unified molecular representation through four-channel feature concatenation and multi-layer perceptron fusion. Molecular structural information is extracted by a high activity aware graph encoder, while simultaneously integrating global descriptors and local atom features that have undergone activity-aware feature projection. An activity-score-dynamically-guided attention fusion mechanism is introduced, enabling the model to prioritize structural features associated with high activity. Finally, an activity-weighted output predictor generates the normalized predicted value (range 0-10).

### 2.1 Feature extraction

#### 2.1.1 Global descriptors

Based on the SMILES representation of molecules, ten key physicochemical descriptors were computed using RDKit package (16). These include molecular weight, LogP, topological polar surface area, number of hydrogen bond donors/acceptors, number of rotatable bonds, number of aromatic rings, fraction of sp³ hybridized carbons (CSP³ ratio), number of heteroatoms, and number of amide bonds (17). These descriptors comprehensively characterize the overall physicochemical properties of molecules at a macroscopic level. After standardization, they are converted into fixed-length numerical vectors, serving as foundational feature inputs for the subsequent model.

#### 2.1.2 Morgan fingerprints

We employed a circular topological environment with a radius of 2 to generate Morgan molecular fingerprints with a length of 1024 bits (18). By encoding the neighborhood structure around each atom within a specific radius, this approach effectively captures key local substructure information within the molecule. The fingerprint algorithm transforms the topological structure of a molecule into a fixed-length binary vector representation, which not only preserves the uniqueness of structural features but also provides discriminative structured inputs for machine learning models.

#### 2.1.3 Local atom features and graph structure construction

Each molecule is represented as a graph structure G = (*V*, *E*), where atoms constitute the node set *V* and chemical bonds constitute the edge set *E*. Each atom node v_i_ ∈ V is characterized by a 6-dimensional feature vector, specifically including: atomic number, atom degree, aromaticity, number of attached hydrogens, formal charge, and whether the atom is in a ring. Chemical bonds are represented as undirected edges, fully preserving the molecular topology. For special cases such as single-atom molecules, self-loop connections are added to ensure graph integrity. This graph representation method provides subsequent graph attention networks with an input that maintains both the completeness of the chemical structure and rich atomic-level local information.

### 2.2 High activity aware graph encoder

This is the core module of this study, and its architecture is illustrated in **Figure 1B**. This module introduces a high activity aware mechanism on the basis of a Graph Attention Network (GAT), achieving enhanced representation of key molecular structures through double-layer attention stacking and feature transformation. Specifically, the encoder first obtains node features via the graph attention layer, then re-weights the features using the high activity aware mechanism, and finally stabilizes the training process through residual connections and normalization operations.

#### 2.2.1 Graph attention network​

For a given molecular graph G=(V,E), let the initial feature matrix of node $v_{i}\in V$ be denoted as $h_{\text{i}}^{\left(l\right)}\in {\mathbb{R}}^{n\times d_{G}}$, where n denotes the number of atoms and $d_{G}$ denotes the initial feature dimension of each atom. N(i) represents its neighboring nodes of the node i. The attention coefficient $e_{ij}^{\left(l\right)}$ between the node i and its neighbor j is first computed as follows Equations 1, 2:

(1) $${\tilde{h}}^{\left(l\right)}=Wh^{\left(l\right)}$$

(2) eij(l)=LeakyReLU(aTh˜i(l)∥h˜j(l)),j∈N(i)

Where W is a trainable weight matrix, $a^{T}$ is the attention parameter vector, and ∥ denotes the concatenation operation. The attention coefficient $\alpha_{ij}$ is then normalized as Equation 3:

(3) $$\alpha_{ij}^{\left(l\right)}=\frac{\exp(e_{ij}^{\left(l\right)})}{\sum_{k\in N(i)}\exp(e_{ik}^{\left(l\right)})}$$

Node features are updated through weighted summation and nonlinear activation Equation 4:

(4) $${\text{h}}_{i}^{\left(l+1\right)}=\sigma (\sum_{j\in \text{N}(i)}\alpha_{ij}{\tilde{h}}_{j}^{\left(l\right)})$$

The final output ${\text{H}}^{\left(l+1\right)}=[{\text{h}}_{1}^{\left(l+1\right)};\ldots ;{\text{h}}_{i}^{\left(l+1\right)}]\in {\mathbb{R}}^{n\times d_{G}}$is obtained with 4 attention heads per GAT layer to capture multi-dimensional feature interactions, further enhancing its representational capacity.

GAT employs an attention mechanism to capture local dependencies within the graph structure. Specifically, during the feature update at the ( l+1)-th layer, the graph attention operation is first applied, followed by a residual connection that effectively alleviates the vanishing gradient problem. Batch normalization is then used to accelerate model convergence and reduce internal covariate shift. The process is detailed as follows Equations 5, 6:

(5) $$H_{\text{res}}^{\left(l+1\right)}=\text{BatchNorm}(H^{\left(l+1\right)}+H^{\left(l\right)})$$

(6) $$H_{elu}^{\left(l+1\right)}=ELU(Dropout(H_{res}^{\left(l+1\right)},p))$$

To further enhance the robustness of the model, a dropout regularization strategy is introduced during the training phase.

#### 2.2.2 High activity aware mechanism

To further enhance the model’s ability to perceive high activity molecules, we introduce a multi-head self-attention module after the standard graph attention layer, the multi-head self-attention module uses 4 attention heads. This module performs global weighting on node features, highlighting molecular substructures associated with high activity. The feature matrix is linearly projected into *query* (Q), *key* (K), and *value* (V) as Equations 7–9.

(7) $$Q_{i}=W_{q}^{i}H_{elu}^{\left(l+1\right)}\in {\mathbb{R}}^{\text{n}\times d_{k}}$$

(8) $$K_{i}=W_{k}^{i}H_{elu}^{\left(l+1\right)}\in {\mathbb{R}}^{\text{n}\times d_{k}}$$

(9) $$V_{i}=W_{v}^{i}H_{elu}^{\left(l+1\right)}\in {\mathbb{R}}^{\text{n}\times dv}$$

Where $W_{q}^{i},W_{k}^{i}\in {\mathbb{R}}^{\text{n}\times d_{k}},W_{v}^{i}\in {\mathbb{R}}^{\text{n}\times d_{v}}$ is the projection weight matrix, $d_{k}$ denotes the *key* dimension, $d_{v}$ denotes the *value* dimension. *i* denotes the *i*-th head. For multi-head attention with *h* heads, the outputs of all heads are concatenated and projected Equations 10–12:

(10) $$Attention\text{(Q,K,V)=Softmax(}\frac{{\text{QK}}^{\text{T}}}{\sqrt{d_{k}}}\text{)V}\in {\mathbb{R}}^{n\times d_{v}}$$

(11) $$head_{i}=Attention(Q_{i},K_{i},V_{i})$$

(12) $$O^{\left(l+1\right)}=Concat(head_{1},\ldots ,head_{h})$$

The output is then combined with the graph attention output $O^{\left(l+1\right)}$, forming high activity aware features that dynamically weight key molecular substructures. Then, to obtain the graph-level embedding representation of the entire molecule, we apply mean pooling to the node representations. We obtain the node feature matrix as $H_{att}^{\left(l+1\right)}\in {\mathbb{R}}^{n\times d_{GAT}}$​( n denotes the number of atoms and $d_{GAT}$ denotes the initial feature dimension of each atom) through GAT operation as Equations 13, 14.

(13) $$H_{att}^{\left(l+1\right)}=BatchNorm(H_{elu}^{\left(l+1\right)}+Dropout(O^{\left(l+1\right)},p))$$

(14) $$H_{\text{graph}}^{\left(l+1\right)}=\frac{1}{n}\overset{n}{\underset{\text{i}=1}{\sum }}H_{att,i}^{\left(l+1\right)}\in {\mathbb{R}}^{1\times d_{GAT}}$$

Where $H_{att,i}^{\left(l+1\right)}$ denotes the feature vector of the *i-*th node. This strategy effectively mitigates overfitting through a random dropout mechanism. Subsequently, after updating all node features, the model obtains the graph-level embedding representation of the entire molecule by computing the average pooling of node representations. This integrated process of dimensionality transformation and the stabilization training strategy work in concert, ensuring both the efficiency and reliability of the model in handling complex molecular graph data.

### 2.3 High activity aware feature projection

The Activity-Aware Feature Projection Module is one of the core components of this study. It adopts a dual-branch parallel processing architecture, as illustrated in **Figure 1C**, consisting of a feature projection branch and an activity-aware gating branch. By introducing an activity score-guided feature gating mechanism, the module achieves adaptive importance weighting of molecular features, effectively enhancing the model’s ability to represent high activity molecules.

#### 2.3.1 Feature projection and gating mechanisms

This dual-branch parallel architecture implements dimensionality transformation through the feature projection branch and feature importance weighting through the activity-aware gating branch Equations 15, 16:

(15) $$\theta =\sigma (W^{'}\cdot \text{GELU}(\text{BatchNorm}(W\cdot \text{Concat}[{\text{H}}_{\text{global}}{\text{,H}}_{\text{local}}{\text{,H}}_{\text{morgan}}]+b))+b^{'})$$

(16) Ga=σ(W′·ReLU(Wθ+b)+b')

Where the feature type ${\text{H}}_{\text{global}}\in {\mathbb{R}}^{\text{B}\times {\text{d}}_{\text{global}}},{\text{H}}_{\text{local}}\in {\mathbb{R}}^{\text{B}\times {\text{d}}_{\text{local}}},{\text{H}}_{\text{morgan}}\in {\mathbb{R}}^{\text{B}\times {\text{d}}_{\text{morgan}}}$(B is the batch size, $d_{global}$, $d_{local}$, and $d_{morgan}$ are the input feature dimension of global descriptors, local atom features, and morgan fingerprints, respectively). Wand $W^{'}$ are the weight matrices. The θ is generated by the activity score estimator in the main model. $H_{pro}$ is the output feature matrix of projection, band $b^{'}$ are the bias terms. The following elaborates on feature projection and gating mechanisms, as well as stabilization training strategies.

We introduce an activity-guided ranking loss, denoted as $L_{rank}$, which aims to construct an explicit supervisory signal that forces the model to learn the rule that compounds with high activity should receive higher feature importance score θ. Specifically, within each training batch, we divide the compounds into a high-activity group $H_{1}$ and a low-activity group $L_{1}$ based on their ground-truth IC_50_. The threshold for grouping can be set according to the data distribution (e.g., the top 30% as high-activity and the bottom 30% as low-activity). Subsequently, the batch-wise averages of the corresponding feature importance scores for the two groups are calculated respectively Equation 17:

(17) $$\theta_{high}=\frac{1}{\left|H_{1}\right|}\sum_{i\in H}\theta^{\left(i\right)},\theta_{low}=\frac{1}{\left|L_{1}\right|}\sum_{j\in H}\theta^{\left(j\right)}$$

We expect the model-learned θ to clearly distinguish compounds with different activity levels, if it satisfies $\theta_{high}\mathrm{``}\theta_{\text{low}}$. To this end, we adopt a Hinge-based ranking loss function Equation 18:

(18) $${ℒ}_{rank}=\max(0,-(\theta_{high}-\theta_{low})+m\arg in)$$

where *margin*​ enforces a minimal, discriminative gap between the average importance scores of the high- and low-activity groups. This loss function is activated only when $\theta_{\text{high}}$ does not exceed $\theta_{\text{low}}$ by at least a *margin*, thereby guiding the model optimization to widen the gap between them Equation 19.

(19) $$H_{pro}=\text{Projection}({\text{H}}_{\text{global}},{\text{H}}_{\text{local}},{\text{H}}_{\text{morgan}})=\text{Dropout}(\text{GELU}(\text{BatchNorm}(W({\text{H}}_{\text{global}},{\text{H}}_{\text{local}},{\text{H}}_{\text{morgan}})+\text{b})))$$

The gating weight Ga∈[0,1] reflects the importance of each feature dimension, enabling the model to adaptively adjust features based on activity levels. The gating weights are then applied to the projected features via element-wise multiplication. To enhance generalization capability, random dropout regularization is incorporated, resulting in the final output Equation 20:

(20) $$H_{feature}=H_{pro}⨀Dropout(Ga,p)$$

Where $H_{feature}$ is the output feature matrix of final feature projection. This design enables the model to prioritize structural features associated with high activity molecules, thereby improving the relevance of the representation. Through the activity-score-guided feature gating mechanism, the module achieves adaptive importance weighting of molecular features, effectively strengthening the model’s capacity to represent high-activity compounds.

#### 2.3.2 Stabilization training strategies

To ensure the stability of the training process, batch normalization is applied after each linear transformation Equation 21:

(21) $$\text{BatchNorm}(x)=\gamma \cdot \frac{x-\mu }{\sqrt{\delta^{2}+\epsilon }}+\beta$$

Where μ and $\delta^{2}$are the mean and variance of the current mini-batch data, γ and β are learnable scaling and shifting parameters, and ϵ is a small constant for numerical stability. This operation accelerates training convergence and reduces internal covariate shift by standardizing each feature dimension of the layer input. On this basis, Dropout regularization is applied after batch normalization and the activation function with a probability p, expressed as Equation 22:

(22) $$\text{Dropout}(x,\;p)=\frac{1}{1-p}\cdot m⨀x$$

Where $m\in {\left\{0,1\right\}}^{d}$is a Bernoulli random mask vector. By randomly zeroing out the outputs of some nodes during training, noise is introduced and the co-adaptation among nodes is disrupted, thereby preventing overfitting. These strategies are tightly integrated into the sequential operations of the feature projection branch. Through the sequential computation and integration​ of ${\text{H}}_{\text{global}},{\text{H}}_{\text{local}},{\text{H}}_{\text{morgan}}$, these strategies work synergistically to ensure training stability and generalization capability of the module during complex feature transformations.”

### 2.4 High activity attention fusion

Serving as the information integration hub of the model, the High-Activity Attention Fusion Module employs a progressive dimensionality reduction strategy for efficient fusion of multi-source information. It provides a unified and optimized molecular representation for downstream prediction tasks, significantly enhancing activity prediction accuracy, as illustrated in **Figure 1D**. The module utilizes a multi-source feature collaborative fusion strategy. By integrating four types of heterogeneous molecular features—graph structural features, global descriptors, local atomic features, and Morgan fingerprints—it fully leverages the complementary nature of these features to strengthen the model’ perception of key structures in high-activity molecules. The module first concatenates these four types of heterogeneous features into a unified feature representation. Then the concatenated feature matrix ${\text{H}}_{concat}\in {\mathbb{R}}^{\text{B}\times {\text{d}}_{\text{hidden}}}$ is Equation 23:

(23) $$H_{concat}=\text{Concat[}H_{graph},H_{feature}]$$

Subsequently, a progressive dimensionality reduction strategy is applied, where features are effectively refined through linear transformation, ultimately restoring the output feature dimension to the original hidden layer size.

### 2.5 IC50 prediction

The prediction layer computes an initial raw estimate $\hat{y}$ via Equation 24, which is subsequently mapped to the range [0,1] through a piecewise normalization function defined in Equation 25. The normalized predicted value ${\hat{y}}_{norm}$ is obtained. Specifically, raw predictions below 4.0 are assigned a normalized value of 0; those between 4.0 and 10.0 undergo linear scaling; and those above 10.0 are truncated to 1. This mapping is calibrated to the practical effective range [4,10] of the IC_50_ metric-the negative logarithm of the half-maximal inhibitory concentration, which covers the majority of pharmaceutically relevant compound activities while excluding low-activity samples and rare ultra-high-activity outliers. Together with an activity-weighting mechanism, this design ensures biological plausibility of the outputs and improves the model’s sensitivity to highly active molecules.

(24) $$\hat{\text{y}}=\text{Dropout}(\text{GELU}(\text{BatchNorm}({\text{W}}_{o}{\text{H}}_{concat}+{\text{b}}_{o})))$$

(25) $${\hat{\text{y}}}_{norm}=\{\begin{array}{ll} 0 & \hat{\text{y}}<4.0\\ \frac{\hat{\text{y}}-4.0}{6.0} & 4.0\leq \hat{\text{y}}\leq 10.0\\ 1 & \hat{\text{y}}>10.0 \end{array}$$

Regarding the model training strategy, a mini-batch gradient descent approach is used in each training epoch. Prior to training, all tensor data is ensured to be located on the GPU device, and label dimensions are verified for consistency to prevent dimension mismatch errors. The mean squared error loss is selected as the loss function for this task, as it is well-suited for continuous-value activity prediction. The specifics are as follows Equations 26, 27:

(26) $${ℒ}_{MSE}=\frac{1}{\text{N}}\sum_{\text{i}=1}^{\text{N}}{\left({\text{y}}_{\text{i}}-{\hat{\text{y}}}_{\text{i}}\right)}^{2}$$

(27) $${ℒ}_{total}={ℒ}_{MSE}+\lambda {ℒ}_{rank}$$

Where N is the batch size, ${\text{y}}_{\text{i}}$ is the true IC_50_ of the *i*-th sample, and ${\hat{\text{y}}}_{\text{i}}$ is the model’s predicted value for the *i*-th sample. The hyperparameter λ is a weighting coefficient that balances the contributions of the two loss terms in the overall objective. It controls the relative importance between the primary regression task—minimizing the Mean Squared Error ( $L_{\text{MSE}}$)—and the auxiliary ranking task, which enforces activity-aware ordering through $L_{\text{rank}}$. The purpose of this balancing is to maximize the model’s final performance on the main regression objective.

During training, a gradient clipping strategy is applied, limiting the gradient norm to a maximum of 1.0 to effectively prevent gradient explosion. Before each optimizer step, gradients are cleared to ensure correct gradient computation. The training loss is accumulated over batches and then averaged, providing stable monitoring of training progress.

### 2.6 Model evaluation

Focusing on the IC_50_ prediction task, this study establishes a multi-level evaluation framework integrating assessment design, metric system construction, comparative method selection, and visualization analysis techniques. We designed a unified model comparator that adopts a stratified evaluation strategy, incorporating four types of Graph Neural Network models (GCN, GAT, GraphSAGE, Graph Transformer) and four classic machine learning models (XGBoost, Random Forest, SVM, MLP). This ensures the diversity and representativeness of the evaluated models. All models are managed through a unified interface and compared fairly under identical data preprocessing pipelines and evaluation standards, effectively mitigating bias introduced by data discrepancies. For our curated dataset, the training and test sets are split in an 8:2 ratio based on Murcko scaffolds. This strategy ensures no overlap in scaffold structures between sets, significantly reduces the risk of data leakage, and maintains consistency with established research practices.

To comprehensively evaluate model performance on the regression task of predicting exact IC_50_ values, we employ MSE (Mean Squared Error) and RMSE (Root Mean Squared Error) to quantify performance, where lower values indicate better performance, and R² (Coefficient of Determination), where a higher value indicates better performance. The specific metric definitions and calculation formulas are as follows Equations 28–30:

(28) $$\text{MSE}=\frac{1}{\text{n}}\sum_{\text{i}=1}^{\text{n}}{\left({\text{y}}_{\text{i}}-{\hat{\text{y}}}_{\text{i}}\right)}^{2}$$

(29) $$\text{RMSE=}\sqrt{\text{MSE}}=\sqrt{\frac{1}{\text{n}}\sum_{\text{i}=1}^{\text{n}}{\left({\text{y}}_{\text{i}}-{\hat{\text{y}}}_{\text{i}}\right)}^{2}}$$

(30) $${\text{R}}^{2}=1-\frac{\sum_{i=1}^{\text{n}}{\left({\text{y}}_{\text{i}}-{\hat{\text{y}}}_{\text{i}}\right)}^{2}}{\sum_{i=1}^{\text{n}}{\left({\text{y}}_{\text{i}}-{\bar{\text{y}}}_{\text{i}}\right)}^{2}}$$

Where n is the sample size, ${\text{y}}_{\text{i}}$ is the true value, ${\bar{\text{y}}}_{\text{i}}$ is the mean of the true values, and ${\hat{\text{y}}}_{\text{i}}$ is the predicted value. The value of R² lies in the range [0,1], with values closer to 1 indicating a stronger explanatory power of the model regarding data variability. In cases where the test set labels exhibit no variability (e.g., zero variance), R² is set to negative infinity to prevent misleading results. Over 50 training epochs, the loss function values (e.g., MSE) on both the training and test sets are recorded simultaneously to analyze model convergence, stability, and generalization ability.

## 3 Results and discussions

### 3.1 Datasets

The dataset in this study was sourced from the ChEMBL (19) and BindingDB (20) databases. We constructed a high-quality dataset comprising 4,856 unique small-molecule compounds by screening for all compounds reported to exhibit inhibitory activity against five key members of the Type III receptor tyrosine kinase (RTK) family: human c-KIT (21), PDGFRα (22), PDGFRβ (23), CSF-1R (24), and FLT3 (25). Each compound is associated with reliable bioactivity data (IC_50_ values). Using the open-source toolkit RDKit (https://www.rdkit.org/docs/index.html), each SMILES descriptor was converted into a molecular graph structure, where nodes represent atoms and edges represent chemical bonds. Both node and edge features were derived from molecular properties. https://www.rdkit.org/.

### 3.2 Baseline methods

To comprehensively evaluate the performance of our proposed model, we compared it with eight competitive baseline methods in the field, comprising four supervised graph neural network methods and four classical machine learning methods. Brief descriptions of these baseline methods are provided below:

Supervised Graph Neural Network Methods: GCN (26), GAT (27), GraphSAGE (28), and Graph Transformer (29).

GCN (Graph Convolutional Network):​ A classic graph neural network based on graph convolution operations, which learns node features by aggregating neighborhood information.

GAT (Graph Attention Network):​ A graph neural network that introduces an attention mechanism, enabling the assignment of differential weights to different neighboring nodes.

GraphSAGE (Graph Sample and AggregatE):​ An inductive graph learning method employing sampling and aggregation strategies, suitable for large-scale graph data.

Graph Transformer:​ A graph neural network based on a self-attention mechanism, capable of effectively modeling long-range dependencies.

Classical Machine Learning Methods: XGBoost (30), Random Forest (31), SVM (32), and MLP (33).

XGBoost (Extreme Gradient Boosting):​ A gradient boosting decision tree model that achieves excellent regression performance through an ensemble learning strategy.

Random Forest:​ A classical method based on decision tree ensembles, known for its good generalization ability and robustness.

SVM (Support Vector Machine):​ A support vector regression model that constructs an optimal hyperplane based on the principle of structural risk minimization.

MLP (Multilayer Perceptron):​ A basic feedforward neural network serving as the fundamental benchmark.

All baseline models were evaluated under a unified experimental setup and common evaluation criteria, using identical training/test dataset splits to ensure comparability and reproducibility. The graph neural network models were implemented based on the PyTorch Geometric framework (34), while the classical machine learning models were built using the scikit-learn and XGBoost libraries. Evaluation metrics included Mean Squared Error (MSE) (35), Root Mean Squared Error (RMSE), and the Coefficient of Determination (R²), providing a comprehensive assessment of model performance from both error measurement and explained variance perspectives. Convergence characteristics and generalization capabilities of each model were analyzed in-depth by examining the loss curves and metric trends over 50 training epochs.

### 3.3 Implementation and hyperparameters

We construct a systematic framework for IC_50_ prediction, comprehensively covering key stages including data preprocessing, molecular representation, model architecture, training strategies, and hyperparameter optimization. The framework is implemented in a GPU environment based on PyTorch, fully leveraging hardware acceleration capabilities to significantly improve computational efficiency. During the data preparation stage, raw molecular data was obtained from the ChEMBL database and subjected to a rigorous standardization pipeline: molecular structures were standardized using RDKit, deduplication was performed based on Morgan fingerprints (radius=2, 1024 bits), and bioactivity data (e.g., IC_50_ values) were filtered and quality-controlled. This resulted in a final set of 4,856 high-quality, structurally unique small-molecule compounds. For molecular representation, an advanced featurizer was employed to represent each compound as a graph structure, with atoms as nodes (with attributes such as atom type and formal charge) and chemical bonds as edges (with bond type and topological information). Furthermore, global physicochemical properties, local atom features, and 1024-bit Morgan fingerprints were integrated to comprehensively capture structural information across multiple scales. The core of the model is the high activity aware graph encoder. Its architecture is based on a two-layer Graph Attention Network (GAT), with 4 attention heads per layer and a hidden dimension of 128. Residual connections were introduced between layers to enhance gradient flow and improve training stability. The feature projection module maps multi-source information—from nodes, edges, global, and local atom features—into a unified 128-dimensional latent space, while the fusion module integrates this information through concatenation and a graph attention mechanism. The final output layer employs a Sigmoid activation function to constrain predictions within a biologically plausible range. The model incorporates a large number of hyperparameters, with the key settings summarized in **Table 1**.

**Table 1:** Hyperparameter used in HGATT.

Hyperparameters	Value
Epochs	50
Learning rate	1e-3
Learning rate decay	0.5
Batch size	16
Loss function	MSE
Dropout	0.3
Optimizer	AdamW
Hidden_dim	128
Weight decay	1e-4
Margin	0.2
λ	0.1

For training, the AdamW optimizer was used in conjunction with a performance-based learning rate scheduler (ReduceLROnPlateau), with Mean Squared Error (MSE) as the loss function. To prevent overfitting, regularization strategies such as Dropout (p=0.3) and gradient clipping (maximum norm of 1.0) were applied. Data splitting followed an 8:2 train/test ratio based on Murcko scaffolds, ensuring no scaffold overlap between sets and effectively mitigating the risk of data leakage. Candidate molecule screening was conducted via a multi-stage process, including preliminary filtering based on IC_50_ thresholds and structural diversity analysis using Murcko scaffolds, ensuring the final selection of the Top 100 candidate molecules covers a wide range of scaffold classes.

For hyperparameter optimization, a grid search was systematically conducted to evaluate the combined effect of hidden layer dimensions (128, 256, 512) and batch sizes (16, 32, 64). The experimental results, as shown in **Table 2**, indicate that model performance does not improve monotonically with increased parameter scale. The optimal configuration was achieved with a hidden dimension of 128 and a batch size of 16 (Model-(128, 16)), yielding the highest R² of 0.5674 and the lowest RMSE and MSE. Specifically, increasing the hidden dimension from 128 to 512 (with batch size fixed at 16) led to a 28.4% decrease in R². Similarly, increasing the batch size from 16 to 64 (with the hidden dimension fixed at 128) resulted in a 17% reduction in R². This suggests that, given the current limited data size (4,856 molecules), excessive model complexity or overly large batch sizes can easily induce overfitting. Conversely, a moderate batch size (e.g., 16) may introduce beneficial gradient noise that acts as a form of regularization, thereby enhancing model robustness. Other hyperparameters, such as the learning rate, dropout rate, and weight decay, were set with reference to common practices and were co-optimized with the architectural parameters. This approach achieved a good balance between training stability and predictive performance.

**Table 2:** Sensitivity analysis of model performance to architectural parameters.

Model	R²	RMSE	MSE
Model (64,16)	0.5174	0.5630	0.3170
Model (64,32)	0.5049	0.5606	0.3141
Model (64,64)	0.4908	0.5641	0.3182
**Model (128,16)**	**0.5674**	**0.5321**	**0.2831**
Model (128,32)	0.4650	0.5948	0.3538
Model (128,64)	0.4707	0.5836	0.3406
Model (256,16)	0.4040	0.6230	0.3881
Model (256,32)	0.4652	0.5948	0.3538
Model (256,64)	0.4955	0.5646	0.3188
Model (512,16)	0.4061	0.6172	0.3810
Model (512,32)	0.4680	0.5764	0.3322
Model (512,64)	0.4335	0.5955	0.3546

Bold values indicate the best performance across all metrics shown.

### 3.4 Performance comparison between HGATT with other approaches

#### 3.4.1 Performance comparison of IC_50_ prediction

**Figure 2:**
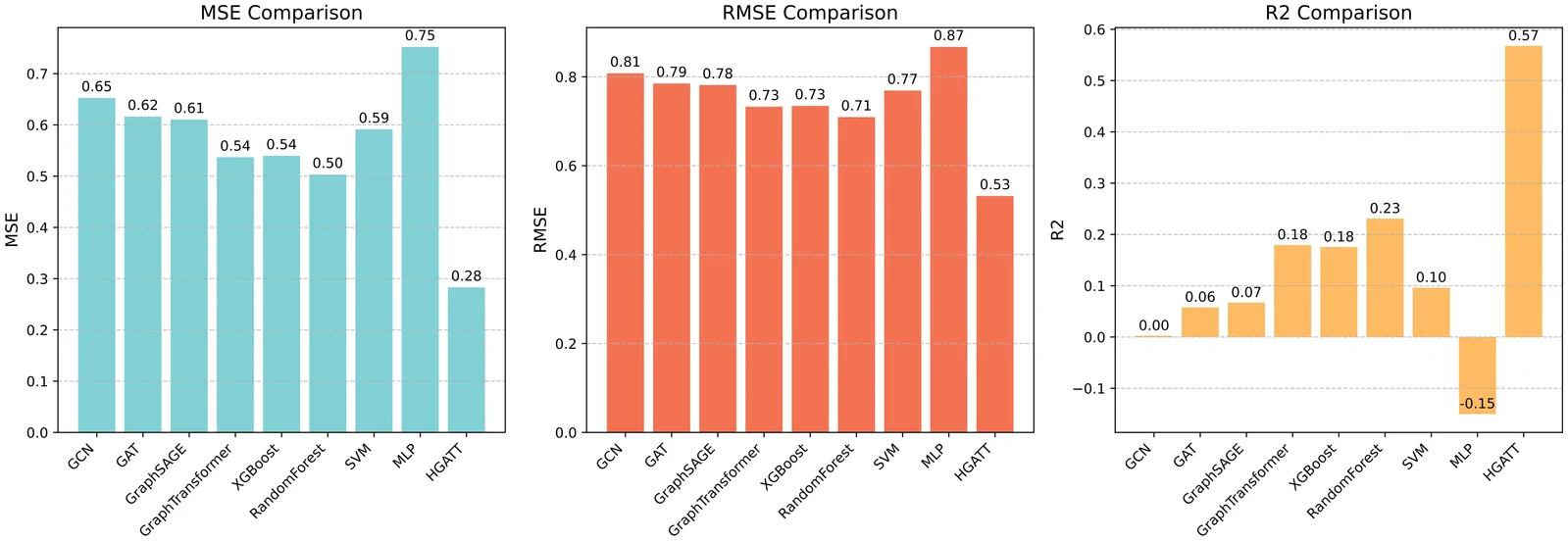
Performance of HGATT with other methods in MSE, RMSE, and R².

To systematically evaluate the effectiveness of the proposed model, this study employed three metrics—Mean Squared Error (MSE), Root Mean Squared Error (RMSE), and the Coefficient of Determination (R²)—and compared HGATT with eight baseline models, including GCN, XGBoost, and Random Forest, under the same dataset and experimental conditions in **Figure 2**. The results show that HGATT significantly outperforms all baseline models across all metrics. Specifically, HGATT achieved a MSE of 0.28, which is 44% lower than that of the second-best model, Random Forest (0.50). This advantage stems from the high activity aware graph encoder, which accurately captures key activity-related regions in molecular structures through a multi-head attention mechanism, thereby improving prediction accuracy.

In terms of RMSE, HGATT reached 0.53, reflecting a 25.35% improvement in stability compared to Random Forest (0.71), indicating that the multi-modal feature fusion strategy effectively enhances model robustness. Notably, in the R² metric, HGATT attained a value of 0.57, the only model to yield a positive score, significantly outperforming all other models (which showed negative values). In the field of cheminformatics, an R² of 0.57 is considered moderate to high, indicating that the model can explain more than half of the variance in the target variable, demonstrating good practical applicability. This is largely attributed to the activity-weighted prediction mechanism, which strengthens the learning of patterns associated with highly active molecules. The unexplained variance may arise from experimental noise, limitations in molecular representation, or unmodeled complex nonlinear relationships.

To further verify the reliability of the results, an independent samples t-test (α = 0.05) was conducted, showing that the performance differences between HGATT and all baseline models are statistically significant (p < 0.01). This confirms that the performance improvement is not due to chance but reflects the inherent effectiveness of the model.

#### 3.4.2 Training analysis and result comparison

**Figure 3:**
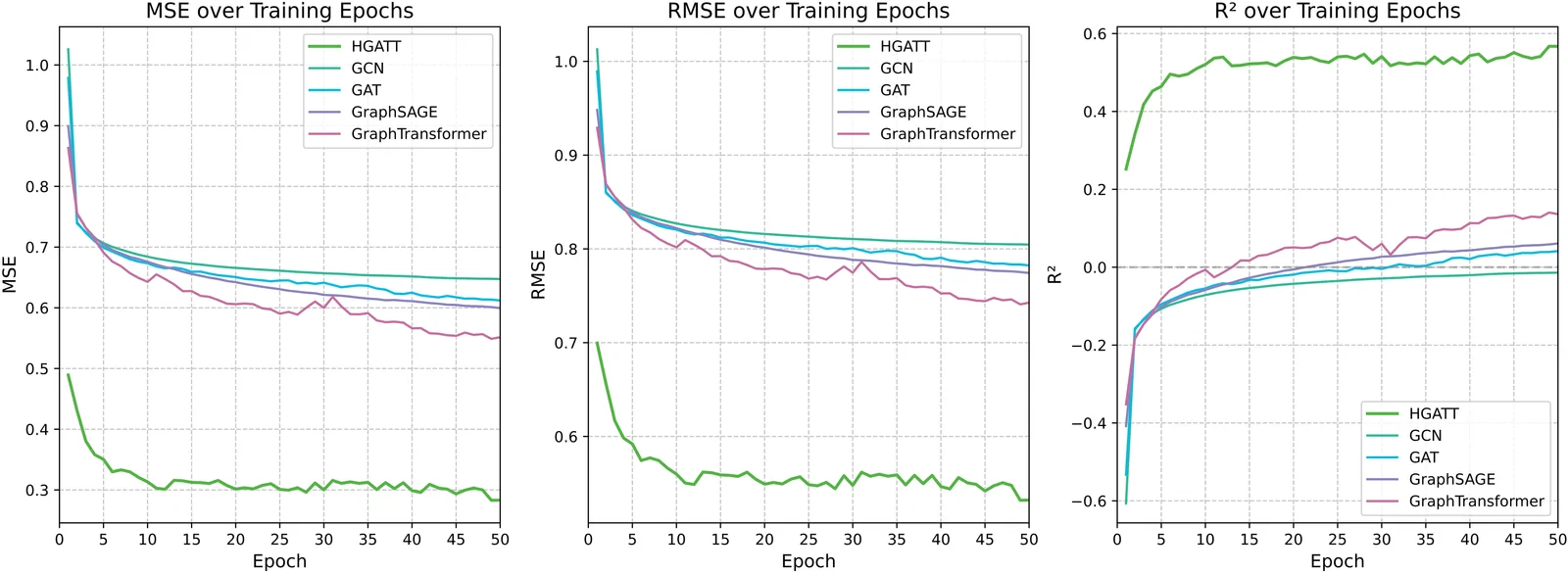
Model performance metrics comparison over training epochs.

**Figure 3** illustrates the variation trends of multiple evaluation metrics for HGATT and baseline models, including GCN, GAT, and GraphSAGE, over 50 training epochs. From the perspective of overall training dynamics, HGATT demonstrates significant advantages across all assessed metrics. In terms of Mean Squared Error (MSE) and Root Mean Squared Error (RMSE), the curve representing HGATT (in green) exhibits the fastest descent rate from the early stages of training, rapidly converging to the globally lowest levels (MSE stabilizes at approximately 0.28, and RMSE at about 0.53). This performance is notably superior to that of other models (e.g., GCN with an MSE around 0.74 and RMSE around 0.86), highlighting its exceptional learning efficiency and predictive accuracy. Regarding the coefficient of determination (R²), which measures the model’s explanatory power, HGATT is the only one that consistently improves and stabilizes within a positive range (around 0.57). In contrast, the R² values of comparative models fluctuate near zero (e.g., GraphSAGE with an R² of approximately -0.25). This outcome strongly indicates that HGATT effectively captures the inherent patterns within the data rather than overfitting to noise.

Further analysis reveals that this superior performance originates from the high-activity-aware graph encoder and the multi-modal feature fusion mechanism employed by the model. These components enable more precise identification of key regions in the molecular structure associated with biological activity, thereby facilitating efficient and stable performance gains during training. In summary, the training dynamics validate the rationality and innovativeness of HGATT’s architectural design, providing process-level evidence for its comprehensive superiority in complex molecular property prediction tasks.

#### 3.4.3 Analyzing model performance through loss convergence curves​

**Figure 4:**
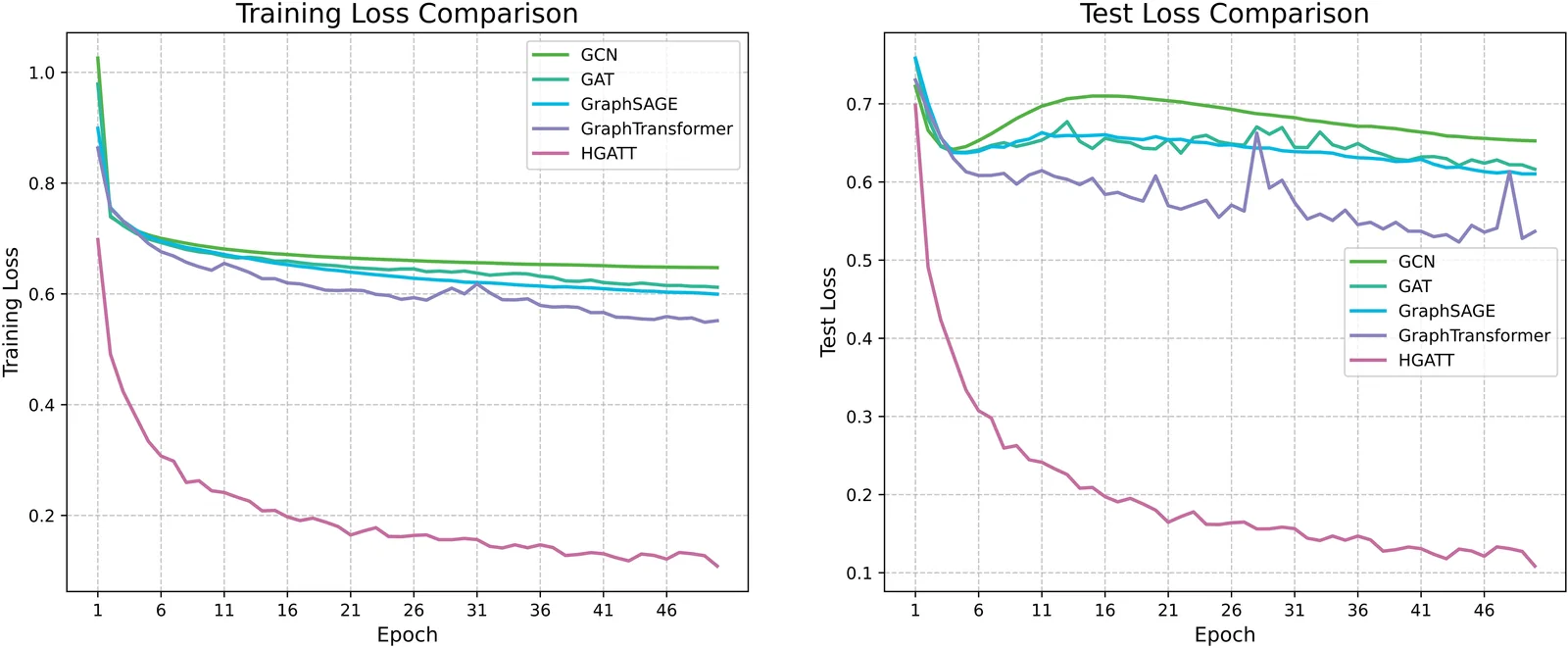
Training and Test Loss Comparison Based on five GNN-based methods.

To quantify the model’s optimization efficiency and generalization capability more deeply, we compare the convergence trajectories of the Mean Squared Error (MSE) loss​ across all models under a unified dataset and identical experimental setting. As shown in **Figure 4**, the loss convergence trajectories of all models over 50 training epochs are plotted on a logarithmic scale.

In terms of training loss, HGATT demonstrates a significant convergence advantage. In the early training stage (Epoch < 6), its loss rapidly decreased from approximately 0.6 to below 0.1, while the losses of GCN and GAT remained above 0.4 during the same period. By the end of training (50 epochs), the loss of HGATT stabilized at an extremely low level of about 0.01, significantly outperforming GCN and GAT (~0.2), GraphSAGE (~0.05), and Graph Transformer (~0.03). The test loss results further validate the superior generalization capability of HGATT. Its test loss curve decreased rapidly with training, eventually converging to the lowest point at approximately 0.02. In contrast, the final test losses for GCN and GAT were as high as ~0.25 and ~0.22, respectively, indicating limited generalization ability. Graph Transformer exhibited a clear tendency of overfitting, with its test loss (~0.08) being significantly higher than its training loss (~0.03). Both the training and test losses of HGATT converged to the lowest levels with a minimal gap, demonstrating not only its strong fitting capacity but also its effective learning of the underlying data patterns, thereby showcasing excellent generalization robustness.

### 3.5 Ablation study

To quantitatively evaluate the independent contributions of each core component, we conducted an ablation study based on the optimal baseline model. Specifically, we designed three ablation variants: (i) removal of the attention fusion module, (ii) removal of the HAA-GAT module, and (iii) removal of the activity gate module. The experimental results, summarized in **Table 3**, demonstrate that model performance declined significantly when the attention fusion module was individually removed, with the R² dropping to 0.4915, the RMSE rising to 0.5750, and the MSE increasing to 0.3392. Performance dropped even more markedly when the HAA-GAT module was separately ablated, with R² decreasing substantially to 0.2253, RMSE rising to 0.6602, and MSE increasing to 0.4822. Similarly, the removal of the activity gate module led to noticeable degradation, where the R² decreased to 0.3202, RMSE increased to 0.6066, and MSE rose to 0.4523, underscoring its crucial role in capturing feature importance to enhance prediction accuracy. These quantitative results indicate that all three modules contribute importantly to model performance, with the HAA-GAT module playing the most critical role. Mechanistically, the HAA-GAT module enhances the recognition of key pharmacophores by prioritizing activity-sensitive molecular regions through its high-activity-aware attention mechanism. The attention fusion module improves feature integration by dynamically aggregating multi-source features and capturing long-range dependencies. The activity gate module further refines predictions by learning to weight important features adaptively. Through these distinct yet complementary mechanisms, the three modules jointly enhance overall model performance.

**Table 3:** Results of the ablation study.

Model	R²	RMSE	MSE
HGATT	**0.5674**	**0.5321**	**0.2832**
HGATT (w/o Attention Fusion)	0.4915	0.5750	0.3392
HGATT (w/o HAA-GAT)	0.2253	0.6602	0.4822
HGATT (w/o Activity Gate)	0.3202	0.6066	0.4523

HAA-GAT, High activity aware GAT.

Bold values indicate the best performance across all metrics shown.

### 3.6 Performance visualization for the top 100 molecules

**Figure 5:**
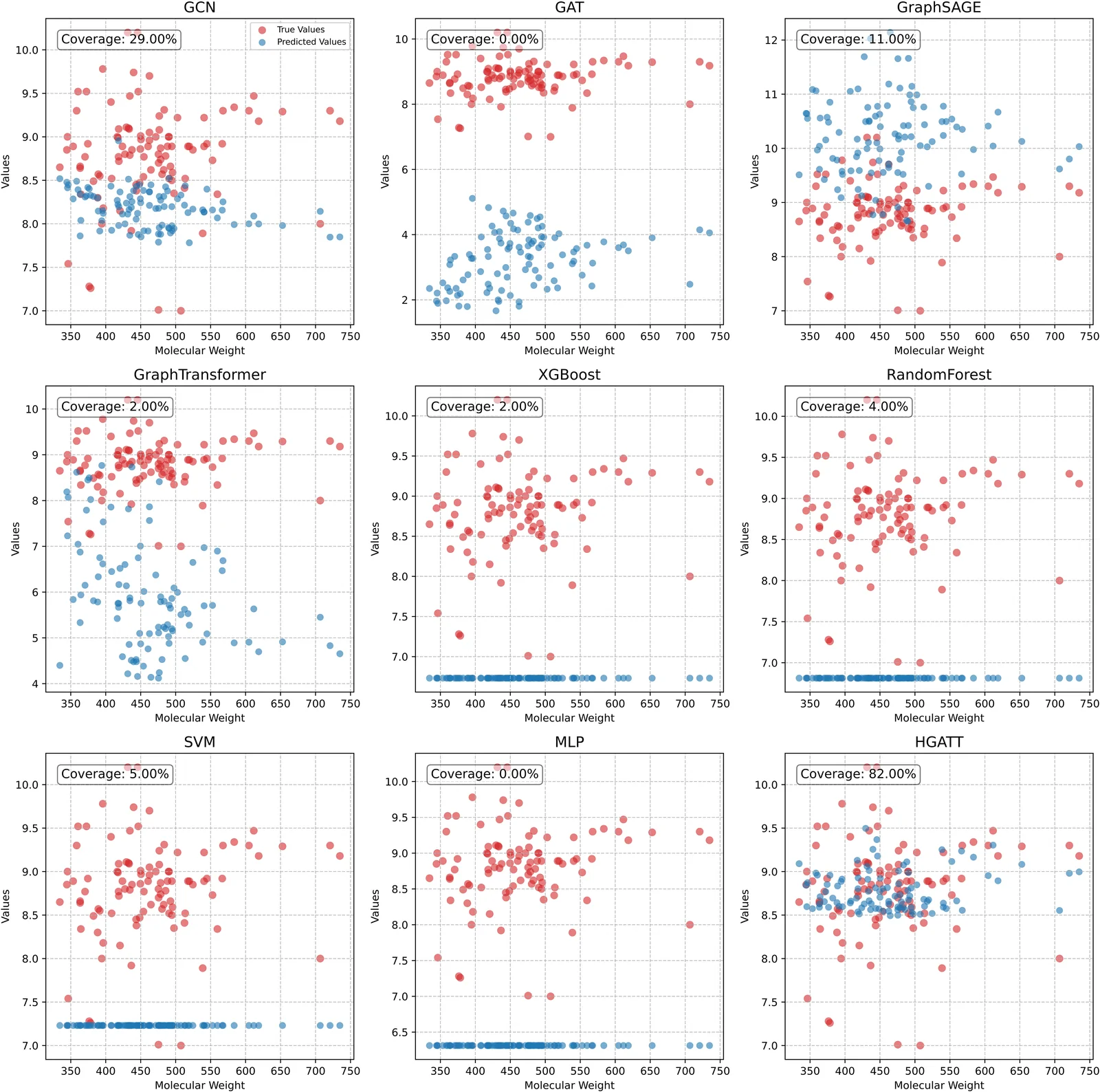
The top 100 molecules on which HGATT and the eight methods performed best on a specific dataset are compared via scatter plots.

To gain a microscopic understanding of the performance limits and reliability of each model on its best-predicted samples, this study selected and compared the top 100 molecules ranked by the smallest absolute error between predicted and true values for each model. As visualized in **Figure 5**, which displays the prediction results of HGATT and the eight baseline models, clear differences in prediction accuracy and consistency can be observed even on the “easiest-to-predict” samples selected by each model.

In the coordinate system with molecular weight (0–650) on the horizontal axis and IC_50_ value (0–10) on the vertical axis, ideally the blue scatter points (predicted values) should completely overlap the red scatter points (true values). However, in most baseline models (e.g., GCN, GAT), while the blue points roughly follow the distribution of the red points, they show noticeable vertical dispersion, reflecting considerable fluctuation in their predictions. In particular, the GCN model achieved a coverage rate of only 29.00%, further indicating its limited prediction stability. In contrast, in the sub-plot corresponding to HGATT, the blue scatter points almost completely overlap the red points, forming an extremely compact distribution band, indicating that its predictions are highly concentrated around the true values. This visual assessment is quantitatively supported by the coverage metric: HGATT achieved a coverage rate of 82.00%, significantly outperforming all other models (all remaining models had coverage rates below 30%).

The strong agreement between visual analysis and quantitative results demonstrates that our method not only leads in macro-evaluation metrics but also exhibits superior prediction consistency and robustness at the sample level, thereby validating its excellent generalization capability in complex molecular property prediction tasks.

To gain a microscopic insight into the prediction limits and reliability of different models, and more critically, to eliminate sample selection bias arising from each model independently picking its own best-performing samples, this subsection employs a unified fixed test set scheme to ensure fair comparison. The detailed experimental logic is as follows: we first rank all molecules in the full test set by the absolute error between predicted and ground-truth IC_50_ values exclusively based on the HGATT model, and select the top 100 molecules with the smallest prediction errors as the shared public test subset. All eight baseline models are then evaluated on this identical set of 100 molecules. All subsequent visualization analyses and quantitative indicators (including coverage rate) are computed uniformly on this shared sample set, which guarantees that the performance comparison across all models is built on exactly the same benchmark and free from selection bias.

The prediction results of HGATT and the eight baseline models are visualized in **Figure 5**. Obvious differences in prediction accuracy and consistency can be observed even on this fixed set of test samples.

In the coordinate system with molecular weight (0-650) on the horizontal axis and IC_50_ value (4-10) on the vertical axis, ideally the blue scatter points (predicted values) should completely overlap the red scatter points (true values). However, in most baseline models (e.g., GCN, GAT), while the blue points roughly follow the distribution of the red points, they show noticeable vertical dispersion, reflecting considerable fluctuation in their predictions. In particular, the GCN model achieved a coverage rate of only 29.00%, further indicating its limited prediction stability. In contrast, in the subplot corresponding to HGATT, the blue scatter points almost completely overlap the red points, forming an extremely compact distribution band, indicating that its predictions are highly concentrated around the true values. This visual assessment is quantitatively supported by the coverage metric: HGATT achieved a coverage rate of 82.00%, significantly outperforming all other models (all remaining models had coverage rates below 30%).

The strong agreement between visual analysis and quantitative results demonstrates that our method not only leads in macro evaluation metrics but also exhibits superior prediction consistency and robustness at the sample level, thereby validating its excellent generalization capability in complex molecular property prediction tasks.

### 3.7 Interpretability and pharmacophore attribution analysis

To verify that HGATT captures the structural determinants of highly active c-KIT inhibitors and provides chemically instructive insights beyond global regression metrics, we conducted a multi-level interpretability analysis. Specifically, we investigated: (i) how reliably the model classifies compounds into discrete activity categories that are meaningful to medicinal chemists; (ii) which pharmacophore classes receive preferential attention in high- versus low-activity molecules; and (iii) how atom-level attention weights are distributed across molecules of varying potency. Collectively, these analyses offer a quantitative assessment of the model’s predictive confidence across different activity regimes, along with an attribution of the substructures that the model associates with potent c-KIT inhibition.

#### 3.7.1 Predictive reliability across activity regimes

**Figure 6:**
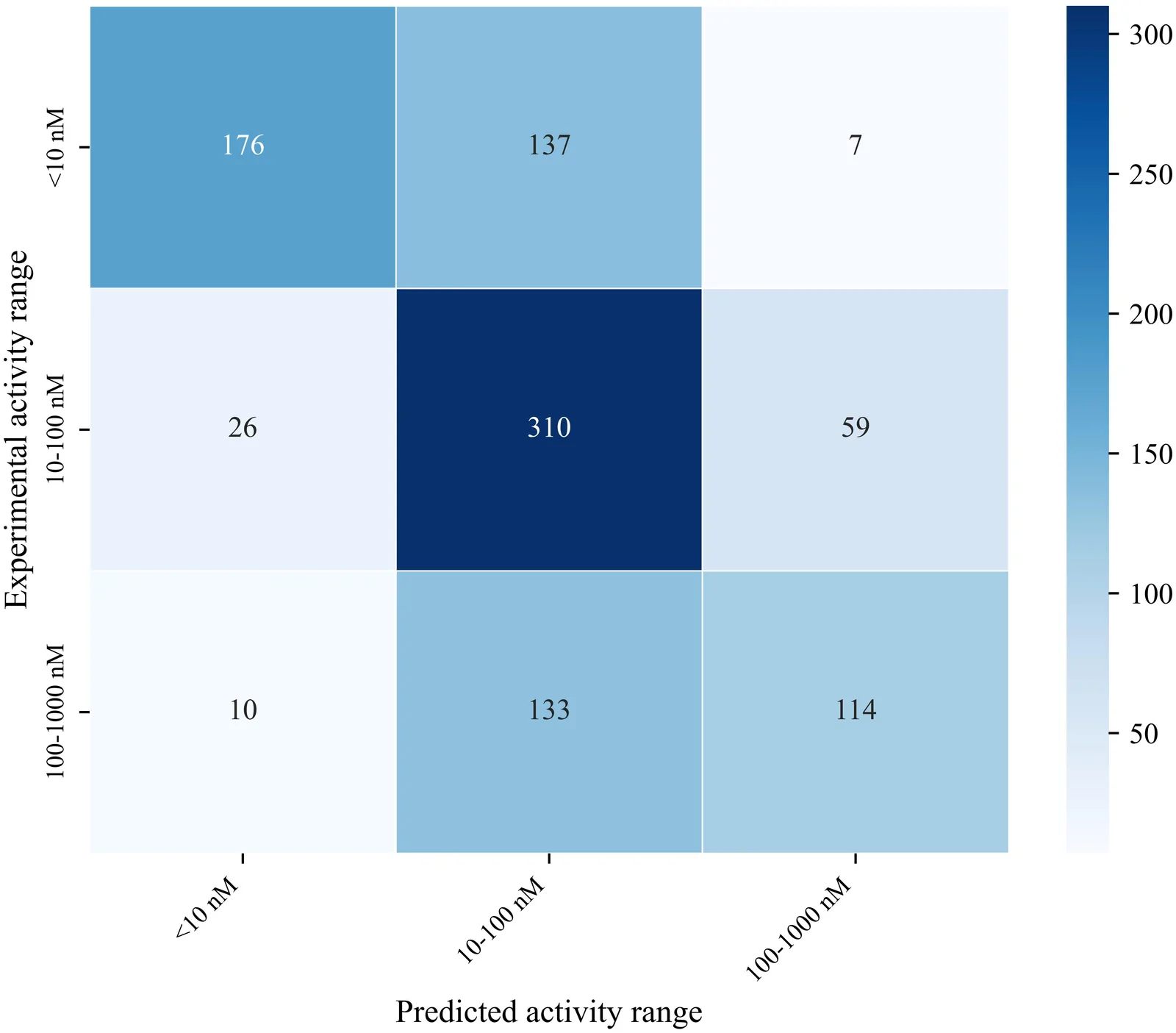
Confusion heatmap of predicted versus experimental activity bins on the independent test set.

Although MSE, RMSE, and R² summarize overall regression accuracy, they fail to reveal whether the model preserves the compound ranking that is most critical in practice, the distinction among highly potent (IC_50_ < 10 nM), moderately active (10–100 nM), and weakly active (100–1000 nM) inhibitors. To address this, we discretized the experimental and predicted pIC_50_ values into three pharmacologically relevant activity bins and constructed a 3 × 3 contingency heatmap (**Figure 6**). On the independent test set (n = 972), the diagonal entries represent the number of compounds correctly assigned to their activity bin, which are 176, 310, and 114 for the high-, medium-, and low-activity bins, respectively. It yields an overall bin-level accuracy of 61.7%.

The model achieved the highest predictive precision for the high-activity category: among the 212 compounds predicted to be highly active, 176 were experimentally confirmed (precision = 83.0%), indicating that the model behaves conservatively when assigning the most valuable activity label. This high precision is particularly desirable in virtual screening, where false positives incur substantial costs. Notably, out of 320 truly highly active compounds, 176 were correctly identified (recall = 55.0%), 137 were predicted as moderately active, and only 7 were misclassified as weakly active. The moderate-activity bin attained the highest recall (78.5%, with 310 out of 395 true moderate compounds correctly classified), and misclassifications predominantly occurred near bin boundaries (e.g., 137 true high-activity compounds predicted as moderate, and 133 true low-activity compounds predicted as moderate), reflecting the continuity of the activity landscape rather than systematic bias. The low-activity bin exhibited a precision of 63.3% (114 correct out of 180 predicted low-activity compounds) and a recall of 44.4%. Compounds are partitioned into high- (<10 nM), moderate- (10–100 nM), and low-activity (100–1000 nM) bins based on their IC_50_ values. Each cell displays the compound count; color intensity scales with the count. The dominant diagonal entries (176, 310, 114) indicate that HGATT preserves pharmacologically relevant compound ordering, achieving particularly high precision (83.0%) for the high-activity category.

**Figure 7:**
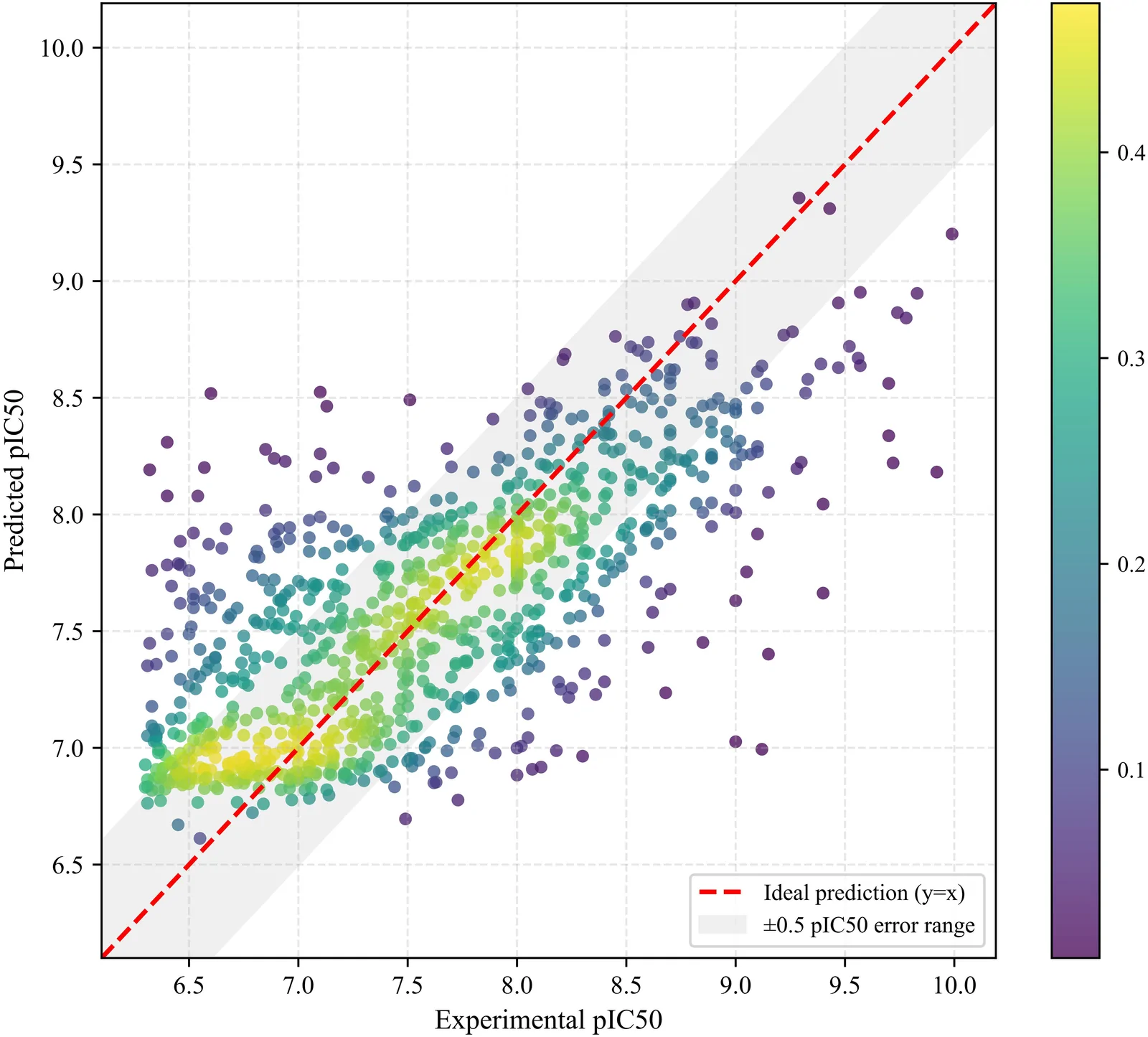
Density-colored scatter plot of predicted versus experimental pIC_50_ on the test set. pIC_50_=-log_10_(IC_50_).

To complement this discrete perspective, **Figure 7** presents the continuous-level prediction results as a density-colored scatter plot of predicted versus experimental pIC_50_, overlaid with the ideal y = x line and ±0.5 pIC_50_ tolerance bands. As shown, the experimental pIC_50_ values span from 6.5 to 10.0, and the predicted values are similarly distributed within the 6.5-10.0 range. The densest region (yellow, local density ≈ 0.4) clusters tightly along the diagonal between pIC_50_ 7.0 and 8.5, where the majority of data points reside and nearly all predictions fall within the ±0.5 tolerance bands. The density color scale transitions from 0.1 (purple, sparse regions) to 0.4 (yellow, dense regions), intuitively reflecting the concentration trend of the data distribution. Predictions remain well-calibrated across the full dynamic range up to pIC_50_ ≈ 9.5, with only modest dispersion observed in the high-activity tail (pIC_50_ > 9.0), where training samples are sparser. This pattern confirms that the bin-level consistency observed in **Figure 6** is not an artifact of discretization but reflects faithful pointwise regression, further supporting the model’s utility in prioritizing the most potent candidates. The red dashed line denotes the ideal prediction line (); the gray shaded band represents the tolerance of pIC_50_ units. Points are color-coded according to local sample density (yellow = high density, purple = low density). The densest cluster lies along the diagonal within the tolerance band, and predictions remain well-calibrated below pIC_50_ ≈ 9.5, confirming reliable pointwise estimation across the activity spectrum.

#### 3.7.2 Pharmacophore-level attribution of high-activity signals

**Figure 8:**
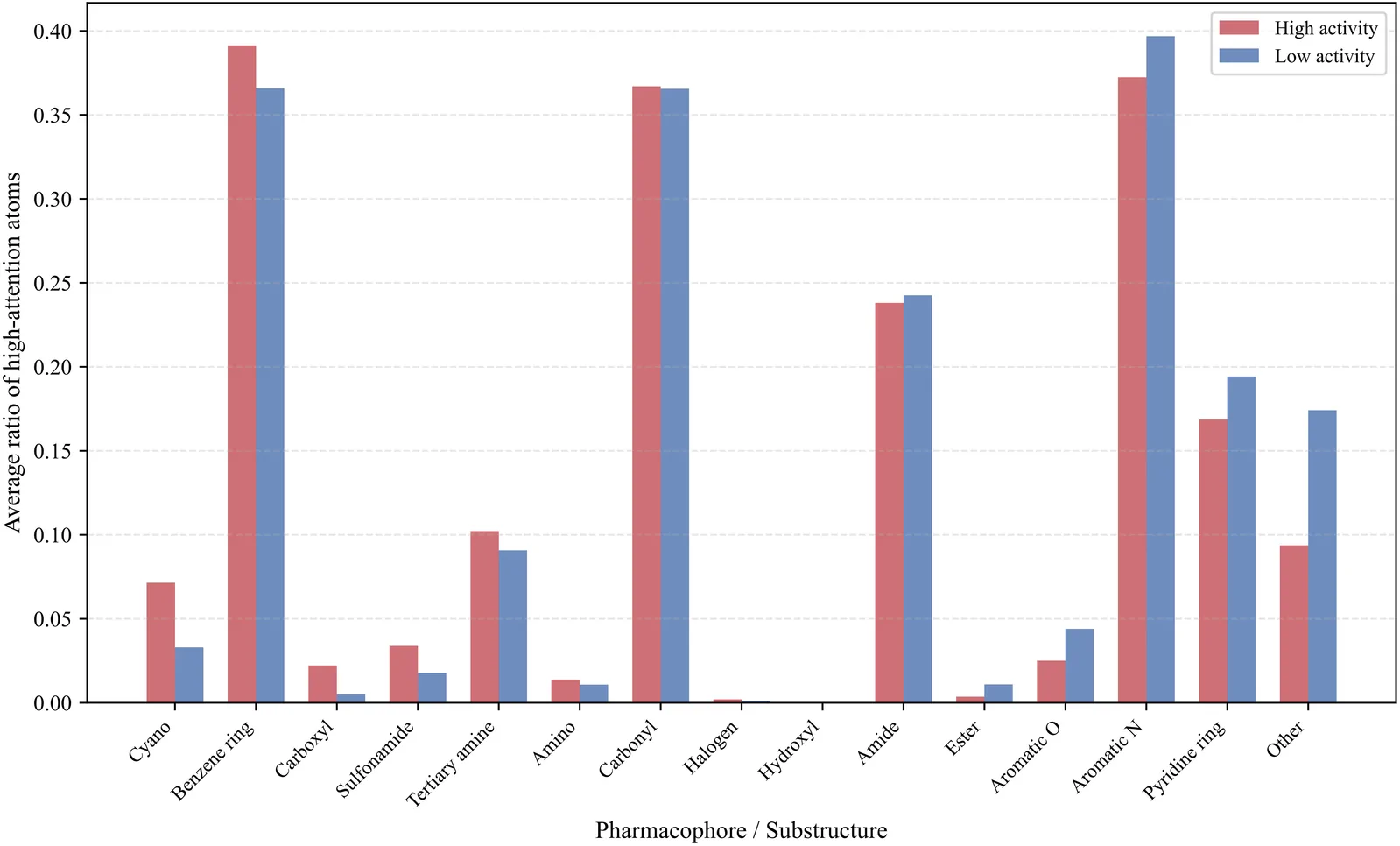
Pharmacophore-level attribution for high-activity versus low-activity molecules.

To identify the substructures preferentially weighted by the high-activity-aware attention mechanism, we aggregated atom-level attention scores by pharmacophore class and compared the average proportion of “high-attention atoms” between the high-activity (IC_50_ < 100 nM) and low-activity (IC_50_ > 100 nM) groups (**Figure 8**). For each molecule, atoms whose normalized attention weights exceeded the 75th percentile were classified as high-attention atoms. The proportion of such atoms was then calculated for each pharmacophore class and averaged across molecules within each activity group. We systematically examined 15 pharmacophore/substructure categories, including cyano, phenyl ring, carboxyl, sulfonamide, tertiary amine, amino, carbonyl, halogen, hydroxyl, amide, ester, aromatic oxygen, aromatic nitrogen, pyridine ring, and ether linkage. Bar plots display the mean proportion of high-attention atoms (normalized attention weights in the upper quartile) within each pharmacophore/substructure category for high-activity (red, IC_50_ < 100 nM) and low-activity (blue, IC_50_ > 100 nM) compounds. Phenyl ring, carbonyl, aromatic nitrogen, and amide constitute a universally weighted core scaffold, whereas the cyano group exhibits a 2.2-fold selective enrichment in high-activity molecules, identifying it as a key structural determinant of potent c-KIT inhibition.

Four pharmacophore classes-phenyl ring, carbonyl, aromatic nitrogen, and amide-received uniformly high attention across both groups (mean proportions ranging from 0.24 to 0.39), indicating that they constitute a general aromatic/hydrogen-bonding scaffold recognized by the model as core binding motifs of type III RTK inhibitors. This is consistent with established c-KIT inhibitor pharmacology, which relies on a heteroaromatic hinge-binding core and amide-linked side chains occupying the ATP-binding pocket. Specifically, the mean high-attention proportions for the phenyl ring (hydrophobic scaffold) were approximately 0.35-0.39, for carbonyl 0.28-0.32, for aromatic nitrogen 0.25-0.30, and for amide 0.24-0.28. These four classes showed minimal variation between high- and low-activity molecules, serving as the foundational pharmacophoric features of c-KIT inhibitors.

Critically, one pharmacophore exhibited a marked and selective enrichment in high-activity molecules: the cyano group (–C≡N). Its high-attention atom proportion was 0.072 in high-activity compounds versus 0.033 in low-activity compounds, representing a 2.2-fold difference—the largest among all examined categories. In c-KIT and related kinase inhibitors, the cyano group frequently functions as a hinge-binding warhead, forming directional hydrogen bonds with the kinase backbone (e.g., Cys673 or the Thr670 gatekeeper region in c-KIT). Its strong selective weighting suggests that HGATT has learned to associate this moiety with enhanced potency rather than treating it as an incidental substituent. Tertiary amines were also slightly favored in high-activity molecules (0.104 vs. 0.091), consistent with their role as solubilizing and cationic center elements in solvent-exposed regions. Conversely, ether linkages and pyridine rings received higher weights in low-activity molecules (ether: 0.095 vs. 0.174; pyridine: 0.170 vs. 0.195), indicating that these features are not diagnostic of high potency by themselves and may, in some contexts, reflect suboptimal substitution patterns. Sulfonamide, carboxyl, ester, aromatic oxygen, amino, hydroxyl, and halogen groups received low attention in both groups (mean proportions all below 0.10), suggesting a relatively minor contribution to the model’s activity decisions for this chemical series.

#### 3.7.3 Atom-level attention distribution reveals multi-pharmacophore synergy

**Figure 9:**
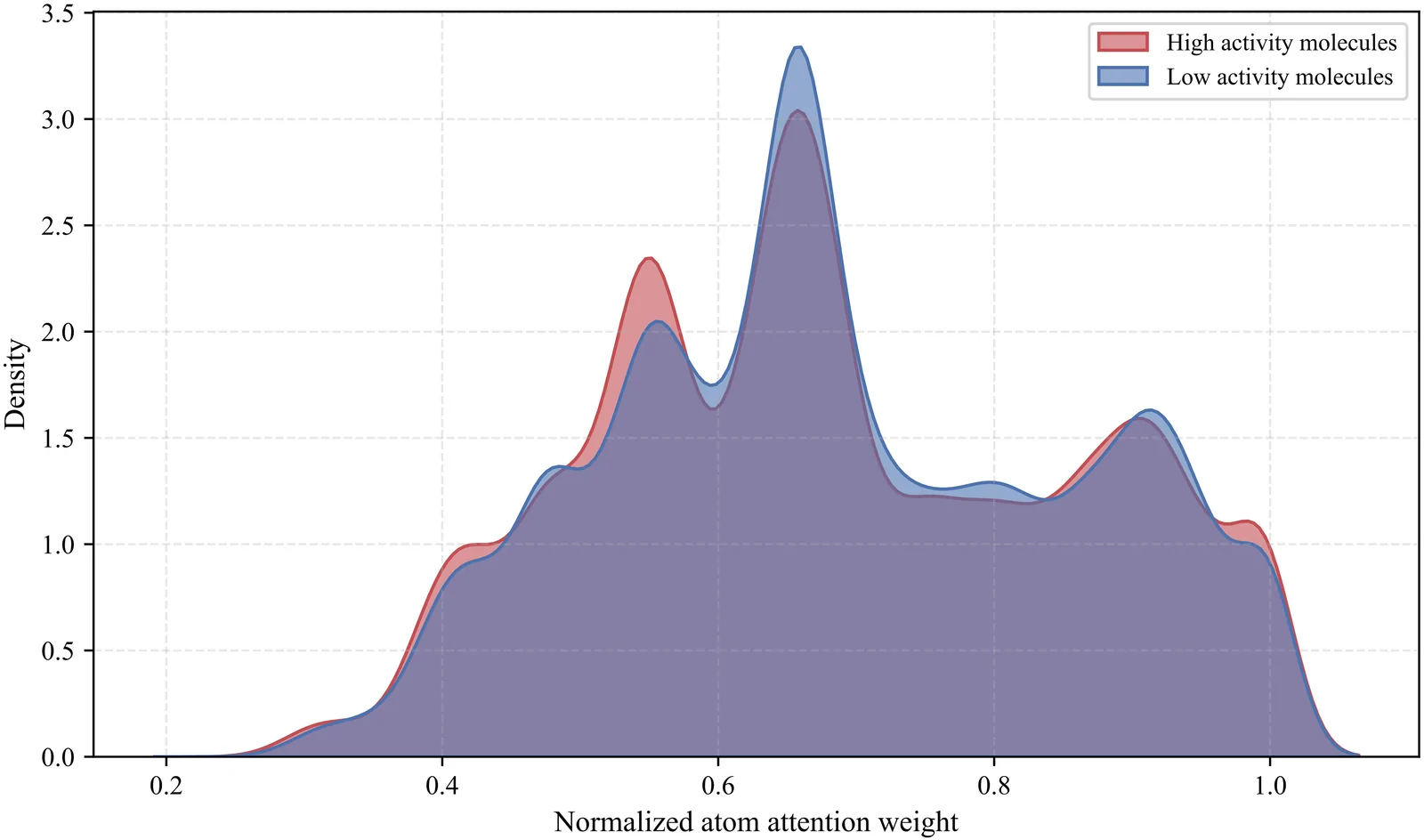
Kernel density estimates of normalized atom-level attention weights for high-activity (red) and low-activity (blue) molecules.

To further characterize the distribution of attention at the atomic level, we computed kernel density estimates (KDE) of the normalized atom-level attention weights separately for high- and low-activity molecules (**Figure 9**). The horizontal axis represents the normalized atom attention weight (range 0.2-1.0), and the vertical axis represents the kernel density estimate (range 0.0-3.5). Both distributions are multimodal, yet their shapes carry chemically informative signatures. Low-activity molecules exhibit a dominant sharp peak at approximately 0.65 (density ≈ 3.3) with a secondary peak near 0.90, indicating that attention is concentrated on a relatively small number of high-weight atoms (i.e., a single dominant substructure) while the remaining atoms receive low weights. In contrast, high-activity molecules retain the peak at 0.65 but with reduced amplitude (density ≈ 3.0), accompanied by a notably enhanced shoulder around 0.55 (density ≈ 2.35) and a small yet distinct peak near 0.95. The net effect is a broader, flatter distribution, with a larger proportion of atoms receiving moderate-to-high attention. Notably, in the intermediate attention range of 0.4-0.6, the density for high-activity molecules is markedly higher than that for low-activity molecules, implying that more atoms participate in contributing to the activity-related signal.

This redistribution is consistent with a multi-pharmacophore model of high potency: potent c-KIT inhibitors do not rely on a single dominant interaction but instead engage the target through a synergistic combination of hinge-binding, hydrogen bonding, hydrophobic, and solvent-exposed elements, each of which must be simultaneously recognized. The high-activity-aware attention mechanism in HGATT appears to capture this synergy by distributing attention more evenly across multiple contributing substructures, rather than collapsing onto a single salient motif. This observation also provides a mechanistic basis for the superior ranking performance reported in Sections 3.4 and 3.6: because high-activity molecules are characterized by a distributed attention signature, the activity-score-driven gating and ranking loss (Section 2.3.1) can learn a global “activity pattern” that is more robust than any single local feature. Low-activity compounds exhibit a dominant peak near 0.65 (density ≈ 3.3), indicating that attention is concentrated on a single predominant substructure. High-activity compounds, in contrast, display a broader, more multimodal distribution with an enhanced shoulder around 0.55 (density ≈ 2.35) and a peak near 0.95, reflecting the multi-pharmacophore synergistic binding that characterizes potent c-KIT inhibitors.

Taken together, the three complementary analyses in this section (bin-level predictive reliability, pharmacophore-level attribution, and atom-level attention distribution) provide concrete, visualizable evidence that HGATT does not merely fit regression targets but genuinely learns chemically meaningful structure–activity relationships. The 2.2-fold selective upweighting of the cyano group and the distributed attention pattern in potent molecules offer testable hypotheses for medicinal chemistry optimization, while the high precision of 83.0% for high-activity classification supports the model’s practical utility as a prioritization tool in virtual screening campaigns targeting c-KIT and related type III RTKs.

## 4 Conclusions

The evaluation of c-KIT inhibitors is crucial for preventing gastrointestinal stromal tumors. Consequently, various machine learning algorithms and molecular fingerprints—such as ECFPs—have been widely adopted for such predictions. However, these methods often fail to fully capture the complex structural and diverse physicochemical characteristics of compounds. To address this limitation, HGATT leverages both the graph structure of molecules and their structural and physicochemical properties as input, and integrates an attention mechanism within a graph attention network (GAT) framework to predict c-KIT inhibitors.

Through the integration of multi-source features and an activity-aware mechanism, we develop a high-activity-aware graph neural network framework. This model demonstrates significant advantages in predicting the IC_50_ of c-KIT inhibitors, achieving a mean squared error (MSE) of 0.28 and a coefficient of determination (R²) of 0.57 on an independent test set, representing a 44% improvement in MSE over the second-best model.​ However, this work has certain limitations, including the impact of the training data size (4,856 molecules) on the predictive stability for rare molecular scaffolds, the limited dimensionality of atomic features that underutilizes quantum chemical or 3D conformational information, and the computational overhead of the multi-module architecture, which is not ideal for real-time large-scale virtual screening.​ Future work can expand in directions such as developing multi-task learning frameworks, integrating generative models, modeling dynamic interactions, and exploring cross-target transfer learning. In summary, the high-performance IC_50_ prediction model constructed in this study provides a reliable tool for targeted drug development, and by incorporating 3D structural information and generative modeling, it offers valuable tools and direction for AI-driven drug discovery.

## Data Availability

The original contributions presented in the study are included in the article/supplementary material. Further inquiries can be directed to the corresponding author.
